# Error management, reliability and cognitive evolution

**DOI:** 10.1007/s10539-017-9583-1

**Published:** 2017-09-11

**Authors:** Bengt Autzen

**Affiliations:** Department of Philosophy, University of Bristol, Cotham House, Bristol, BS6 6JL UK

**Keywords:** Cognitive evolution, Decoupled representation, Error rates, Reliability

## Abstract

The paper offers a partial vindication of Sterelny’s view on the role of error rates and reliability in his theory of decoupled representation based on modelling techniques borrowed from the biological literature on evolution in stochastic environments. In the case of a tight link between tracking states and behaviour, I argue that in its full generality Sterelny’s account instantiates the base-rate fallacy. With regard to non-tightly linked behaviour, I show that Sterelny’s account can be vindicated subject to an adequate evolutionary model and a suitable notion of reliability.

## Introduction

A central problem in evolutionary biology is why some organisms have evolved the ability to mentally represent their environment and to use these internal representations to guide their behaviour. This issue is particularly puzzling since many organisms show behavioural plasticity, that is, the ability to vary their behaviour depending on the environment, without such an elaborate cognitive architecture. Godfrey-Smith (1996) makes the case that the function of cognition is to deal with environmental complexity. That is, a cognitive architecture that includes internal representations is needed in order for organisms to behave adaptively across a wide range of novel environments.

A variant of this argument is found in Sterelny (2003) who emphasises the complexity of social environments in cognitive evolution. More generally, Sterelny develops a theory of decoupled representation in his treatment of the evolution of animal cognition. Decoupled representations contrast with tracking states that are triggered by environmental cues but are only capable of inducing one type of behaviour. Decoupled representations are ‘belief-like’ since they are not associated with particular behaviours but rather assist in prompting different behaviours depending on the context.

Sterelny’s account conjectures a difference between the evolution of tracking mechanisms that are tightly linked with behavioural consequences and those that are not: while natural selection can favour mechanisms with higher error rates if tracking states are tightly linked to behavioural consequences, this selective pressure ceases when tracking states are not tightly linked with behaviour and more reliable mechanisms are favoured. In Sterelny’s (2003, 34) own words:

[S]election can favor a mechanism with a higher error rate over a mechanism with a lower error rate, so long as the higher rate mechanism makes cheap rather than expensive errors. However, I conjecture that this is true only of special-purpose tracking mechanisms—for cognitive systems where the tracking system drives a specific type of behavior. […] As a tracking state ceases to be tightly coupled to a specific behavior, there ceases to be reason to protect against false positives at the expense of false negatives or vice versa. […] [I]f selection on a lineage favors the evolution of capacities for forming decoupled representation, it will favor more reliable mechanisms over less reliable ones.

The aim of this paper is to assess Sterelny’s view on the role of error rates and reliability in cognitive evolution by means of modeling techniques borrowed from the biological literature on evolution in stochastic environments. As such, the paper can be seen as an exercise in error management theory understood as a general logic of decision making under uncertainty when the fitness costs of false positives and false negatives are different (Johnson et al. 2013). In the case of a tight link between tracking states and behaviour, I will argue that in its full generality Sterelny’s account instantiates the base-rate fallacy and make a proposal of how to fix the problem. With regard to non-tightly linked behaviour, I will show that Sterelny’s account can be vindicated subject to an adequate evolutionary model and a suitable notion of reliability.

The structure of the paper is as follows. In a first step, I examine the evolution of decoupled representation when behaviour is tightly linked with tracking states. In a second step, I turn to the case of non-tightly linked behaviour. In a third step, I reconsider the notion of reliability at play in Sterelny’s account.

## Tightly linked behaviour

In order to assess Sterelny’s view on the role of error rates and reliability in cognitive evolution, I will break it down into two propositions. The first proposition refers to the case of a tight link between tracking states and behavioural consequences, the second one to the case of a non-tight link between these variables. The case of a tight link includes both what Sterelny refers to as detection systems and robust tracking systems. A detection system is a tracking system that connects a specific environmental signal with a specific adaptive response (Sterelny 2003, 14). For instance, in order to escape from predatory toads cockroaches detect their presence by means of the wind gust caused by the striking toad’s head. A robust tracking system, in contrast, is not triggered by a single cue (Sterelny 2003, 27–29). For instance, in order to deal with brood parasites reed warblers use multiple cues, such as egg size, timing and cuckoo sightings, to determine whether to accept or to reject eggs in their nest. A central role in the evolution of these different tracking systems is played by the environment according to Sterelny. Detection systems work well in transparent environments, that is, environments that are characterised by simple and reliable correspondences between sensory cues and functional properties. In contrast, detection systems tend to work less well in translucent environments, that is, environments in which functionally similar items have different sensory profiles and functionally different items have very similar sensory profiles. While robust tracking systems do not rely on single cues, these tracking systems are still linked with a specific behavioural response. As such, they fall under the category of a tight link between tracking states and behaviour.

As we have seen, Sterelny (2003, 43) asserts in the case of tightly linked behaviour that “selection can favor a mechanism with a higher error rate over a mechanism with a lower error rate, so long as the higher rate mechanism makes cheap rather than expensive errors”. Since a mechanism can generate both false positives and false negatives, it is unclear what is meant by referring to a mechanism with “a higher error rate”. Are we talking about the false positive or the false negative rate? Consider the following example. Mechanism $M_{1}$ has a false positive rate of 0.05 and a false negative rate of 0.1 while mechanism $M_{2}$ has a false positive rate of 0.1 and a false negative rate of 0.05. While mechanism $M_{1}$ fares better with regard to false positives, mechanism $M_{2}$ has a lower false negative rate.

Despite the slightly ambiguous wording, I assume that Sterelny has something like the comparison of mechanisms $M_{1}$ and $M_{2}$ in mind when discussing tracking states with tightly linked behavioural consequences. The claim then is that selection can favour a mechanism with a higher false positive error rate (such as $M_{2}$) over a mechanism with a higher false negative rate (such as $M_{1}$) if and only if the behavioural consequences of false positives have lower fitness costs than the consequences of false negatives (analogously, selection can favour mechanism with a higher false negative rate over a mechanism with a higher false positive rate if and only if the behavioural consequences of false negatives have lower fitness costs than the consequences of false positives). Let us denote this first claim as proposition $P_{1}$.

In order to study proposition $P_{1}$ more systematically, I will introduce a simple formal model describing a population whose members differ with regard to their cognitive mechanisms. The initial model has four different variables: environmental states *S*, tracking states *T*, behavioural consequences *C* and fitness payoffs *V*. To begin with, I assume that variable *S* can take the two different values $S_{1}$ and $S_{2}$ and might indicate whether or not a predator is present. Tracking states are denoted by the variable *T*. Again, I assume that the variable *T* can take two different values. Tracking state $T_{1}$ indicates that the predator is present while $T_{2}$ indicates that it is not. Finally, I assume that there are two possible behavioural consequences: either the individual tries to escape ($C_{1}$) or stays put and continues foraging ($C_{2}$). Each behavioural consequence is associated with a fitness payoff conditional on the environmental state faced by an individual, which is denoted by $V_{ik}$ ($i,k \in \{1,2\}$). For instance, the term $V_{21}$ denotes the payoff associated with choosing behaviour $C_{1}$ when the environmental state is $S_{2}$. In line with the general view that false negatives are more troublesome than false positives for biological defence mechanisms, such as fight-or-flight responses, immune reactions or coughing, it is assumed that the fitness payoffs of false negatives are significantly lower than the fitness payoffs of false positives (i.e., $V_{12} < V_{21}$) (Nettle 2012, 72). For ease of computation it is assumed that the fitness payoffs $V_{11}$ and $V_{22}$ are numerically identical. Finally, it is assumed that making errors is more costly than not making errors (i.e., $V_{21} < V_{11}$).

In order to describe how cognitive mechanisms link environmental states with behavioural responses, I have to introduce some probabilistic structure. First, there is the conditional probability distribution of tracking states given environmental states. For instance, $P(T_{1}|S_{1})$ denotes the probability of adopting tracking state $T_{1}$ when the environmental state is $S_{1}$. Of course, the larger $P(T_{1}|S_{1})$, the more accurate the cognitive mechanism. In contrast, $P(T_{2}|S_{1})$ denotes the false negative rate, that is, the probability of adopting the false tracking state $T_{2}$ if the environmental state is $S_{1}$. Analogously, $P(T_{1}|S_{2})$ denotes the false positive rate of the cognitive mechanism.

Having introduced a conditional probability distribution of tracking states given environmental states, more probabilistic structure is needed in order to fully describe the cognitive architecture linking environmental states with behavioural consequences. To do so, I introduce a further conditional probability distribution assigning probabilities to behavioural consequences given tracking states. For instance, $P(C_{1}|T_{1})$ denotes the probability of choosing behavioural consequence $C_{1}$ based on having tracking state $T_{1}$. As the current focus is on tightly linked behaviour it is assumed that $P(C_{1}|T_{1}) =P(C_{2}|T_{2}) = 1$ and $P(C_{2}|T_{1}) =P(C_{1}|T_{2}) = 0$.

Since the scenario of a tight link between tracking states and behavioural consequences is supposed to cover both the cases of detection systems and robust tracking systems, the model is indifferent to whether a tracking state is driven by a single cue or by multiple cues in a given state of the world. The environmental states denoted by variable *S* will generally come in a more fine-grained description in the case of a robust tracking system compared to the case of a detection system. A further methodological point worth mentioning is that the distinction between tracking states and behavioural consequences is somewhat redundant in this scenario since tracking states are tightly linked with particular behavioural consequences. The reason for adapting this richer model structure right from the start is that it allows a smoother transition to modeling the case of a non-tight link between tracking states and behaviour later on.

Next, it is assumed that that all population members experience independent environmental states. For instance, if the state variable *S* describes the presence (or absence) of a predator, the independence assumption entails that an independent random experiment decides whether an individual encounters a predator with probability $P(S_{1})$. Phrased differently, predation risk is idiosyncratic; it independently affects the members of the population. This assumption makes sense if, for instance, uncoordinated predators attack members of a highly scattered population. Further, it is assumed that organisms independently adopt a tracking state given an environmental state. This assumption captures the idea that idiosyncratic factors affect an organism’s perception of its environment.

A cognitive mechanism can be seen as a strategy that specifies the probability of a behavioural consequence given an environmental state. In this model of organisms facing idiosyncratic risk the appropriate fitness measure for such a strategy is given by the expected fitness payoff (McNamara 1995):

1 $$\begin{aligned} P(S_{1})[P(T_{1}|S_{1})V_{11} + P(T_{2}|S_{1})V_{12}] + P(S_{2})[ P(T_{1}|S_{2})V_{21} + P(T_{2}|S_{2})V_{22}]. \end{aligned}$$

Having established a formal measure based on which selection chooses among cognitive mechanisms, I can now return to proposition $P_{1}$. While Sterelny is right that evolution can favour mechanism $M_{2}$ over mechanism $M_{1}$, it is false to assert that this requires the fitness payoff of false positives to be larger than the payoff of false negatives (or, alternatively, the fitness costs of false positives to be smaller than the costs of false negatives). Focusing exclusively on the fitness payoffs (or, alternatively, fitness costs) ignores the crucial role of the probability of state $S_{1}$ in expression (1). In fact, a low fitness payoff of false positives can be compensated by a large value of probability $P(S_{1})$. Phrased informally, if predators are rare, then one has to worry less about the fitness impact of false negatives.

To illustrate, consider the mechanisms $M_{1}$ and $M_{2}$ introduced earlier. To recapitulate, mechanism $M_{1}$ has a false positive rate of $P(T_{1}|S_{2}) = 0.05$ and a false negative rate of $P(T_{2}|S_{1}) = 0.1$ while mechanism $M_{2}$ has false positive rate of $P(T_{1}|S_{2}) = 0.1$ and a false negative rate of $P(T_{2}|S_{1}) = 0.05$. The following fitness payoffs are chosen in line with the previous qualitative assumptions: $V_{11}= 1$, $V_{12}= 0.1$, $V_{21}=0.5$ and $V_{22}= 1$. In a first scenario it is assumed that a predator is equally probable to be present or to be absent, that is, $P(S_{1})= P(S_{2})=0.5$. By applying criterion (1), mechanism $M_{1}$ has an expected fitness payoff of 0.943 and mechanism $M_{2}$ has an expected fitness payoff of 0.953. Hence, mechanism $M_{2}$ is favoured over mechanism $M_{1}$ by natural selection.

So far, the numerical example has been in line with proposition $P_{1}$. Now, consider a second scenario in which predators are assumed to be very rare, that is, $P(S_{1})=0.01$. Again, applying expression (1) yields an expected fitness payoff of 0.974 for mechanism $M_{1}$ and an expected fitness payoff of 0.950 for mechanism $M_{2}$. Selection now favours mechanism $M_{1}$ over mechanism $M_{2}$ even though the fitness payoffs associated with the different error probabilities remain unchanged compared to the previous example.

Proposition $P_{1}$ in its full generality is wrong because it ignores the ‘base rates’ of the environmental states. Ignoring information about base rates in an inference situation is generally referred to as the ‘base-rate fallacy’. The base-rate fallacy has many manifestations.^1^ While proposition $P_{1}$ instantiates this probabilistic fallacy, matters can be fixed and, hence, Sterelny’s position vindicated if we presume that all environmental states are equally probable. In that case, selection favours mechanisms with a higher false positive rate over mechanisms with a higher false negative rate if and only if the behavioural consequences of false negatives have lower fitness costs than the consequences of false positives.

An alternative analysis might adopt a more liberal notion of a fitness payoff (or fitness cost) associated with false positives and false negatives. In particular, one might include the probability of an environmental state into the definition of a fitness payoff. Formally, the payoff associated with a false positive could be defined as $V_{21}^{*}=V_{21}P(S_{2})$ and the fitness payoff of a false negative as $V_{12}^{*}=V_{12}P(S_{1})$.^2^ Doing so has the virtue of rendering proposition $P_{1}$ true. However, this proposal not only has the drawback that its notion of a fitness payoff of an error rate is not very intuitive but also, and more importantly, it sits uneasily with standard treatments of error management theory found in the biological literature. For instance, Nettle (2012) separates the fitness payoffs from the probabilities of environmental states. I therefore consider the more liberal notion of fitness payoffs as an unsatisfactory formal analysis of Sterelny’s account.

The present model can also be used to illustrate and assess some other assertions found in the philosophical literature on naturalistic theories of representation.^3^ For instance, Artiga (2013) discusses the error management of cockroaches and crickets. These organisms possess a set of slender filiform hairs at the rear of their abdomen that are sensitive to air movement. When these appendages detect some high speed air movement, they trigger a range of behaviours in their hosts aimed at evading predators. Artiga (2013, 269) suggests that these appendages “probably most of the time” trigger an evasive behaviour when there is no predator around. Formally speaking, the quantity of interest is the probability of the environmental state that no predator is present given that the cockroach performs an evasive behaviour, that is, $P(S_{2}|T_{1})$.^4^ By applying Bayes’ theorem (as well as the law of total probability), this probability can be calculated as

$$\begin{aligned} \frac{P(T_{1}|S_{2})P(S_{2})}{P(T_{1}|S_{1})P(S_{1})+P(T_{1}|S_{2})P(S_{2})}. \end{aligned}$$

Suppose that the error rates of the cockroach’s cognitive mechanism are identical to those of mechanism $M_{1}$ introduced earlier. Now, a number of scenarios can be considered with regard to the probability of environmental states. Let us start with assuming that predators are very rare, that is, $P(S_{1}) = 0.01$ (and, hence, $P(S_{2}) = 0.99$). In that case the probability that a predator is present given that the cockroach performs an evasive behaviour is approximately 0.16. Phrased differently, given that the cockroach has a neuronal state that triggers evasive behaviour, its neuronal state is false approximately 84% of the time. This result is very much in line with Artiga’s view. Matters are different, however, when encounters with predators are much more common. Suppose that the probability of a predator being present is equal to 0.5 (i.e., $P(S_{1}) = 0.5$). In that case, the probability that the predator is present given that the cockroach performs an evasive behaviour is approximately 0.95. That is, given that the cockroach has a neuronal state that triggers evasive behaviour, its neuronal state is false approximately only 5% of the time. Given the numerical assumptions regarding the error rates of mechanism $M_{1}$, the threshold for having a true neuronal state most of the time if the cockroach performs an evasive behaviour is approximately $P(S_{1})=0.05$. Phrased differently, if the probability of a predator being present sinks under 0.05 it is more probable than not that the cockroach has a false neuronal state given its neuronal state triggers an evasive action.

Returning to the main discussion, it was so far assumed that organisms face idiosyncratic risk. In an alternative scenario all organisms in the population face the same environmental state. That is, rather than performing an independent random experiment for each organism determining whether, say, an organism faces a predator, a single random experiment now determines the environmental state affecting the whole population. Phrased differently, the risk faced by the organisms becomes aggregate.^5^ As an illustration, one might think about the population being attacked by a large group of predators. Or alternatively, the organisms might face an environmental hazard such as a thunderstorm that might flood the population’s habitat or set it on fire. In that case the fitness measure has to be modified. Rather than using the expected fitness payoff, geometric mean fitness or, equivalently, expected logarithmic fitness sets the right standard for determining evolutionary success (McNamara 1995):

2 $$\begin{aligned} P(S_{1})\log [P(T_{1}|S_{1})V_{11} + P(T_{2}|S_{1})V_{12}] + P(S_{2})\log [ P(T_{1}|S_{2})V_{21} + P(T_{2}|S_{2})V_{22}]. \end{aligned}$$

While the quantitative details of the example change due the adoption of a different fitness measure, the qualitative point remains unaffected. Again, the probabilities of the environmental states are relevant for determining the evolutionary success of a cognitive mechanism.

## Non-tightly linked behaviour

I will now turn to the case where tracking states are not tightly linked with behavioural consequences and assess the claim, here denoted as proposition $P_{2}$, that selection will favour more reliable mechanisms over less reliable ones. Understand proposition $P_{2}$ requires a closer look at the notion of reliability. Sterelny does not elaborate on this concept but refers to the work of Godfrey-Smith for a more detailed discussion. Godfrey-Smith (1996) proposes a two-dimensional notion of reliability. In order to assess the reliability of a tracking mechanism, both its false positive rate and its false negative rate have to be taken into account. Importantly, Godfrey-Smith does not offer a measure of reliability that tells us how to weigh false positive and false negative rates in order to combine them into a single reliability score. In a sense the two different error rates are incommensurable. Returning to the tracking mechanisms $M_{1}$ and $M_{2}$, Godfrey-Smith’s account does not allow us to speak of one of the two mechanisms being more reliable. Focusing on one dimension—false positives—mechanism $M_{1}$ comes out on top and focusing on the other dimension—false negatives—mechanism $M_{2}$ is more reliable. End of story.

Since Sterelny refers to the reliability of a mechanism rather than the reliability of a mechanism *along one dimension*, the question remains of how to interpret this terminology. A natural extension of Godfrey-Smith’s notion of reliability suggests itself, in order to speak of the reliability of a tracking mechanism. If a tracking mechanism is more reliable in one dimension than the other tracking mechanism and at least as reliable in the second dimension as its competitor, then it seems natural to say that the former mechanism is overall more reliable than the latter. Of course, the use of this notion of reliability is limited since it does not apply to pairs of tracking mechanisms that have conflicting reliability orderings depending on the type of error rate in focus. As a consequence Sterelny’s account remains silent in these kind of cases of conflict. Nevertheless, this constrained notion of reliability provides a suitable starting point for assessing proposition $P_{2}$.

In order to model the case of a non-tight link between tracking states and behavioural consequences, the notion of the response breadth of a cognitive mechanism needs further elaboration. Since in the case of decoupled representation tracking states are not tightly linked to a specific behavioural response, decoupled representation goes along with an increase of response breadth of a cognitive mechanism. Indeed, Sterelny (2003, 34) refers to decoupled representation as being “nothing but very broad-banded response”. The response breadth is understood as the capacity of a cognitive mechanism to respond in more than one way to a feature of the organism’s environment. As such, there is no simple function between the registration of that environmental feature and the behavioural response. Rather, a behavioural response is selected based on what else the organism notices and knows. Phrased differently, the behavioural response is conditional on what I will call the background information of the organism.^6^

Since the evolution of decoupled representation goes along with an increase in the range of possible behaviours, a new model with a larger set of possible behavioural consequences needs to be introduced. To do so, I modify the basic model discussed in the previous section by considering *n* rather than only two possible behavioural consequences. In analogy to the previous model, these behavioural consequences will be denoted as $C_{k}$ with $k \in \{1,2,\ldots, n\}$. The fitness payoff of behavioural consequence $C_{k}$ given environmental state $S_{i}$ is denoted as $V_{ik}$ for $i \in \{1,2\}$ and $k \in \{1,2,\ldots,n\}$. Modeling a broad-banded response also requires the introduction of an additional variable, denoted as *B*, representing the background information of an organism. It is assumed that *B* can take *m* different values, which are denoted by $B_{l}$ with $l \in \{1,2,\ldots,m\}$.

While there is no simple function between a salient feature of the environment, such as the presence of a predator or the occurrence of a thunderstorm, and a behavioural consequence, the tracking variable *T* and background information *B* jointly determine a behavioural consequence. That is, the probability of the behavioural consequence $C_{k}$ given tracking state $T_{k}$ and background information state $B_{l}$, $P(C_{k}|T_{j},B_{l})$ is either 0 or 1. A tight link between tracking state and behaviour was characterised in terms of the probability of choosing a specific behaviour given a tracking state, $P(C_{k}|T_{j})$. In particular, it was assumed that these probabilities are equal to 0 or 1 in the case of a tight link. In the context of non-tightly linked behaviour, no assumptions about the numerical value of these probabilities have been made yet.

For the moment I assume that the value of the background information *B* is sampled independently for each organism. This assumption implies that there is no communication between the members of the population with regard to their background information. The probability of the behavioural response $C_{k}$ given tracking state $T_{j}$ can then be calculated by means of the law of total probability:

$$\begin{aligned} P(C_{k}|T_{j}) = \sum _{l=1}^{m} P(C_{k}|T_{j},B_{l})P(B_{l}). \end{aligned}$$

I assume that the probabilities $P(C_{k}|T_{j})$ are strictly smaller than 1 for all choices of *k* and *j*. This amounts to the fairly sensible assumption that there is a state of the background information *B* that has non-zero probability and will not lead to behavioural response $C_{k}$ given that state $T_{j}$.

In order to analyse the evolution of decoupled representation, I will divide the set of possible behaviours into two subsets. The first subset, denoted as $C_{A} = \{C_{1}, C_{2}, \ldots, C_{\mu }\}$ contains all kinds of behavioural consequences aimed at avoiding a predator (or at seeking refuge from a thunderstorm) while the second subset, denoted as $C_{NA} = \{C_{\mu +1}, C_{\mu +2}, \ldots, C_{n}\}$, contains all kinds of responses not aimed at avoiding a predator. Behaviours of this kind are highly diverse and can include choosing an epistemic action such as staying put and gathering more information. In the revised model I assume that given tracking state $T_{1}$ it is more probably to choose an ‘avoidance’ than a ‘non-avoidance’ behaviour. I assume, however, that even non-avoidance behaviour has a non-zero probability of being chosen given tracking state $T_{1}$. A simplifying assumption is that every avoidance response is equally probable given a tracking state. Similarly, a non-avoidance response is more probable than a non-avoidance response give tracking state $T_{2}$ and each individual non-avoidance behaviour is equally probable given a tracking state.

With regard to the fitness payoffs of the individual behaviours, it is again assumed that false negatives are more costly than false positives, that is, $V_{1h} < V_{2k}$ for any $k \in \{1,2,\ldots,\mu \}, h \in \{\mu +1,\mu +2,\ldots,n\}$. In order to simplify the model, I assume further that for a given tracking state, any avoidance (non-avoidance) behavioural consequence leads to the same fitness payoff. For instance, given tracking state $T_{1}$ any behavioural consequence in the subset $C_{A}$ will have an identical fitness payoff.

In a sense the model is reduced to the basic model describing tightly linked behaviour by dividing the set of possible behavioural consequences into two subsets and by assuming that within a subset all members are equally probable given a tracking state and have identical fitness payoffs given a state of the world. For the purpose of this analysis, one can then represent the set $C_{A}$ by means of a single behavioural consequence $C_{1}$ mimicking the fitness payoffs and conditional probabilities associated with a random member of $C_{A}$. Analogously, one can represent set $C_{NA}$ by means of a single behavioural response $C_{2}$ having identical fitness payoffs and being associated with identical conditional probabilities as a random member of $C_{NA}$. The difference to the model describing tightly linked behaviour is that in the current version neither behavioural response $C_{1}$ is tightly linked with tracking state $T_{1}$ nor is behavioural response $C_{2}$ tightly linked with tracking state $T_{2}$.

Assuming that the organisms face idiosyncratic risk renders the expected fitness payoff as the right fitness measure. In this model the expected fitness payoff of a cognitive mechanism is given by:

3 $$P(S_{1} )[P(T_{1} |S_{1} )[P(C_{1} |T_{1} )V_{{11}} + P(C_{2} |T_{1} )V_{{12}} ]{\text{ }} + P(T_{2} |S_{1} )[P(C_{1} |T_{2} )V_{{11}} + P(C_{2} |T_{2} )V_{{12}} ]]{\text{ }} + P(S_{2} )[P(T_{1} |S_{2} )[P(C_{1} |T_{1} )V_{{21}} + P(C_{2} |T_{1} )V_{{22}} ]{\text{ }} + P(T_{2} |S_{2} )[P(C_{1} |T_{2} )V_{{21}} + P(C_{2} |T_{2} )V_{{22}} ]].$$

In order to address the question of whether selection favours more reliable mechanisms over less reliable mechanisms, let us consider two cognitive mechanisms that differ only with regard to their false negative rates. The question then becomes whether the mechanism with the lower false negative rate will be favoured by selection. It can be shown that the expected fitness payoff is monotonically increasing in $P(T_{1}|S_{1})$ (see “Appendix 1”). That is, improving the reliability of a tracking mechanism by increasing $P(T_{1}|S_{1})$ and, hence, reducing the false negative rate $P(T_{2}|S_{1})$ leads to an increase in the expected fitness payoff. So, under the assumptions of this model, proposition $P_{2}$ turns out to be true.

The discussion vindicates Sterelny’s claims regarding the role of error rates and reliability in cognitive evolution. The vindication is only partial, however, since it is sensitive to the empirical assumptions of the underlying evolutionary model. To illustrate its limitations, consider a rather extreme modification of the model describing a non-tight link between tracking states and behaviour associated with fitness measure (3). Suppose that given a tracking state each possible behavioural consequence $C_{k}$ (with $k \in \{1,2,\ldots,n\}$) is equally probable. More formally, $P(C_{k}|T_{j}) = \frac{1}{n}$ for any $k \in \{1,2,\ldots,n\}$ and $j \in \{1,2\}$. Given these assumptions, the expected fitness payoff of a cognitive mechanism is given by:

4 $$\begin{aligned} \frac{1}{n}P(S_{1})\sum _{k=1}^{n}V_{1k} + \frac{1}{n}P(S_{2})\sum _{k=1}^{n}V_{2k}. \end{aligned}$$

Importantly, the error rates do not figure in this expression. As a result there is no selective pressure on reliability in this scenario. The reason for this result is, of course, the indifference assumption regarding the conditional probabilities of behavioural consequences given tracking states. Since every behaviour is equally probably given a tracking state, it does not matter how accurate the tracking mechanism represents the world. As a result proposition $P_{2}$ is false in this case of non-tightly linked behaviour.

While the indifference assumption underlying fitness measure (4) is clearly unrealistic, the example still illustrates an important point. The link between tracking states and behavioural consequences can counteract any fitness gains due to a higher reliability of a cognitive mechanism. Reliability describes the relationship between tracking states and states of the world, while fitness is determined by the cognitive architecture connecting environmental states, tracking states, background information and behavioural states. It should therefore not come as a surprise that the truth of proposition $P_{2}$ is sensitive to the empirical assumptions of the underlying biological model.

It might be tempting to characterise the difference between tightly and non-tightly linked behaviour by stating that while selection favours more reliable mechanisms over less reliable mechanisms in the case of non-tightly linked behaviour, selection can favour less reliable mechanisms over more reliable mechanisms in the case of tightly linked behaviour. However, such an assertion is false twice over. For one reason, the case of non-tightly linked behaviour covers the case in which an organism is indifferent regarding the behavioural consequence given a tracking state. However, in that case there is no selective pressure for reliability. For another reason, selection favours more reliable over less reliable mechanisms in the case of tightly linked behaviour (in that case the probabilities $P(C_{1}|T_{1})$ and $P(C_{2}|T_{2})$ are both set equal to 1). Indeed, the selective pressure for reliability, as measured by the difference in fitness values of cognitive mechanisms, decreases when moving from a tight to a non-tight link between tracking states and behaviour (see “Appendix 2”).

So far, it was assumed that the environmental state *S* as well as the background information *B* are sampled independently for each organism. Now, suppose that each organism faces the same environmental state and shares the same state of the variable describing the background information. In this model the members of the population face aggregate risk and share their background information. In this case the fitness measure of a cognitive mechanism is given by

5 $$\sum\limits_{{l = 1}}^{m} P (S_{1} ,B_{l} )\log [P(T_{1} |S_{1} )[P(C_{1} |T_{1} ,B_{l} )V_{{11}} + P(C_{2} |T_{1} ,B_{l} )V_{{12}} ] + P(T_{2} |S_{1} )[P(C_{1} |T_{2} ,B_{l} )V_{{11}} + P(C_{2} |T_{2} ,B_{l} )V_{{12}} ]]{\text{ }} + \sum\limits_{{l = 1}}^{m} P (S_{2} ,B_{l} )\log [P(T_{1} |S_{2} )[P(C_{1} |T_{1} ,B_{l} )V_{{21}} + P(C_{2} |T_{1} ,B_{l} )V_{{22}} ] + P(T_{2} |S_{2} )[P(C_{1} |T_{2} ,B_{l} )V_{{21}} + P(C_{2} |T_{2} ,B_{l} )V_{{22}} ]].$$

This measure offers a formal criterion for studying whether, all other things being equal, improving the reliability of a tracking mechanism by increasing $P(T_{1}|S_{1})$ and, hence, reducing the false negative rate $P(T_{2}|S_{1})$ leads to an increase in fitness in the revised model. Again, an answer to this question will generally depend on the numerical values of the model parameters.

## Reliability reconsidered

A further characteristic of the analysis is the restricted notion of reliability that only allows for a partial ordering of mechanisms according to their reliability. Remember that a mechanism $M_{1}$ was considered as more reliable than a mechanism $M_{2}$ if and only if $M_{1}$ has a strictly smaller error rate than $M_{2}$ along one dimension and an error rate that is smaller than or equal to the error rate of $M_{2}$ along the other dimension. While this constrained account of reliability sits well with Sterelny’s (and Godfrey-Smith’s) work, one might ask whether a more general notion of reliability can be adopted that applies to cognitive mechanisms with different reliability rankings along the two different dimensions.

One proposal of that kind measures the reliability of a mechanism by means of the linear combination of the two error probabilities. A motivation for this reliability measure can be found in statistics. Statisticians have developed test procedures that minimise the linear combination of error probabilities of a hypothesis test (e.g., DeGroot and Schervish 2002, 464). As such, the linear combination of error probabilities is considered as a suitable way of measuring the reliability of a test. Using the linear combination of error probabilities as a reliability measure raises the question of how to weigh the individual error probabilities when forming a linear combination. A natural choice seems to be to assign equal weight to the two different error probabilities. However, it is also conceivable to assign unequal weights to the different error probabilities in order to prioritise one type of error, say false negatives over the other. Indeed, the Neyman–Pearson theory of hypothesis testing prioritises the error probability of incorrectly rejecting a true null hypothesis by requiring that in an optimal test this probability has to be smaller than some fixed value while simultaneously minimising the error probability of incorrectly accepting a false null hypothesis (Neyman and Pearson 1933).

Another proposal that prioritises one error rate over the other makes use of a lexicographic ordering. Suppose a lexicographic reliability ordering prioritises the rate of false negatives. As a result a mechanism is considered as more reliable if and only if its false negatives rate is strictly smaller than the false negatives rate of a competing mechanism. Given the extreme weight put on one type of error in a lexicographic ordering, the implication of this notion of reliability is that proposition $P_{2}$ turns out to be false. To illustrate, consider the following pair of mechanisms. Mechanism $M_{1}$ has a false negative rate of $P(T_{2}|S_{1})=0.06$ and a false positive rate of $P(T_{1}|S_{2})=0.05$, while mechanism $M_{2}$ has a false negative rate of $P(T_{2}|S_{1})=0.05$ and a false positive rate of $P(T_{1}|S_{2})=0.5$. By applying a lexicographic ordering that prioritises false negative rates, mechanism $M_{2}$ is considered as more reliable than mechanism $M_{1}$. Applying fitness measure (3) results in mechanism $M_{1}$ having an expected fitness of 0.81 while mechanism $M_{2}$ having an expected fitness of 0.75. As such, natural selection will favour the less reliable mechanism $M_{1}$.

This result crucially depends on the choice of a reliability ranking for the two mechanisms. While the particular choice of a lexicographic ordering prioritising false negative rates can rightly be questioned, a more general lesson seems to be uncontroversial: not only does the truth of Sterelny’s view on the role of error rates and reliability in cognitive evolution depend on the specific assumptions of the biological model but also on the choice of a suitable notion of reliability. As such, the vindication of Sterelny’s account of decoupled representation is only partial for empirical and conceptual reasons.

## Conclusion

The paper examined come central claims regarding the role of error rates and reliability in Sterelny’s theory of decoupled representation based on a simple model of evolution in stochastic environments. The analysis distinguished between tightly linked and non-tightly linked behaviour. With regard regard to tightly linked behaviour, I argued that in its full generality Sterelny’s account instantiates the base-rate fallacy. In order to avoid the problem, the probabilities of environmental states have to be taken into account. With regard to non-tightly linked behaviour, I offered partial support for the view that selection will favour more reliable tracking mechanisms over less reliable ones. The vindication of Sterelny’s account of decoupled representation offered here is only partial since it relies on the assumptions of the underlying evolutionary model and a suitable notion of reliability of a tracking mechanism.

## Appendix 1

Suppose that a mechanism $M_{1}$ is considered as more reliable than a mechanism $M_{2}$ if and only if $M_{1}$ has a strictly smaller error rate than $M_{2}$ along one dimension and an error rate that is smaller than or equal to the error rate of $M_{2}$ along the other dimension. Without loss of generality suppose that two mechanisms assign the same numerical value to $P(T_{2}|S_{2})$ but differ with regard to the numerical value of $P(T_{1}|S_{1})$. I will show that expression (3) is monotonically increasing in $P(T_{1}|S_{1})$. Since $P(T_{1}|S_{1})$ only figures in the first summand of expression (3), we can focus exclusively on this term. In order to simplify the notation, let us denote the conditional probability $P(T_{1}|S_{1})$ by variable *x*. The first summand of expression (3) then reads as follows

$$\begin{aligned} f(x):=P(S_{1})\Big [x\big [P(C_{1}|T_{1})V_{11} + P(C_{2}|T_{1})V_{12}\big ] + (1-x)\big [P(C_{1}|T_{2})V_{11} + P(C_{2}|T_{2})V_{12}\big ]\Big ]. \end{aligned}$$

In order to show that function *f* is monotonically increasing in *x*, let us consider the first derivative of *f*:

$$\begin{aligned} f'(x)=P(S_{1})\Big [\big [P(C_{1}|T_{1})V_{11} + P(C_{2}|T_{1})V_{12}\big ] - \big [P(C_{1}|T_{2})V_{11} + P(C_{2}|T_{2})V_{12}\big ]\Big ]. \end{aligned}$$

The large bracket on the right hand side can be rewritten as

$$\begin{aligned} \big [P(C_{1}|T_{1})V_{11} + (1-P(C_{1}|T_{1}))V_{12}\big ] - \big [(1-P(C_{2}|T_{2}))V_{11} + P(C_{2}|T_{2})V_{12}\big ]. \end{aligned}$$

Reordering this expression yields

$$\begin{aligned} \Big [\big [P(C_{1}|T_{1})+ P(C_{2}|T_{2})\big ]V_{11} + V_{12} \Big ]- \Big [\big [P(C_{1}|T_{1})+ P(C_{2}|T_{2})\big ]V_{12} + V_{11}\Big ]. \end{aligned}$$

Since $P(C_{1}|T_{1})+ P(C_{2}|T_{2})$ is assumed to be strictly larger than 1 and $V_{11}$ is assumed to be larger than $V_{12}$, we conclude that $f'(x)$ is strictly positive for all values of *x* (under the assumption that $P(S_{1})$ is strictly positive).

## Appendix 2

Suppose that a mechanism $M_{1}$ is considered as more reliable than a mechanism $M_{2}$ if and only if $M_{1}$ has a strictly smaller error rate than $M_{2}$ along one dimension and an error rate that is smaller than or equal to the error rate of $M_{2}$ along the other dimension. Without loss of generality suppose that two mechanisms assign the same numerical value to $P(T_{2}|S_{2})$ but differ with regard to the numerical value of $P(T_{1}|S_{1})$. In particular, assume that mechanism $M_{2}$ is more reliable than mechanism $M_{1}$. I will demonstrate that changing from tightly linked behaviour to non-tightly linked behaviour weakens the selective pressure for reliability measured by the difference in fitness values of the two mechanisms.

In order to simplify the discussion I will introduce the following notation:

6 $$\begin{aligned} \alpha = P(C_{1}|T_{1})V_{11} + P(C_{2}|T_{1})V_{12}, \end{aligned}$$

7 $$\begin{aligned} \beta = P(C_{1}|T_{2})V_{11} + P(C_{2}|T_{2})V_{12}, \end{aligned}$$

8 $$\begin{aligned} \delta = P(C_{1}|T_{1})V_{21} + P(C_{2}|T_{1})V_{22}. \end{aligned}$$

9 $$\begin{aligned} \gamma = P(C_{1}|T_{2})V_{21} + P(C_{2}|T_{2})V_{22}. \end{aligned}$$

Using the parameters $\alpha , \beta , \delta$ and $\gamma$ the fitness measure (3) can then be stated as

$$\begin{aligned} P(S_{1})\Big [P(T_{1}|S_{1}) \alpha + P(T_{2}|S_{1}) \beta \Big ] + P(S_{2})\Big [P(T_{1}|S_{2}) \delta + P(T_{2}|S_{2}) \gamma \Big ]. \end{aligned}$$

Given the assumptions about the fitness payoffs in the model associated with this fitness measure (i.e., $V_{12}<V_{21}<V_{11}=V_{22}$), the following strict inequalities hold if tracking states are non-tightly linked with behaviour (i.e., $P(C_{1}|T_{1}) < 1$ and $P(C_{2}|T_{2}) < 1$):

10 $$\begin{aligned} \alpha < V_{11}, \end{aligned}$$

11 $$\begin{aligned} \beta > V_{12}, \end{aligned}$$

12 $$\begin{aligned} \delta > V_{21}, \end{aligned}$$

13 $$\begin{aligned} \gamma < V_{22}. \end{aligned}$$

Let $p_{1}$ denote the probability $P(T_{1}|S_{1})$ of mechanism $M_{1}$ and $p_{2}$ denote the same probability of mechanism $M_{2}$. The difference in fitness values between mechanism $M_{2}$ and mechanism $M_{1}$ can then be stated as

$$\begin{aligned} ( \alpha - \beta )(p_{2}-p_{1}). \end{aligned}$$

In the case of a tight link between tracking state and behaviour this reduces to

$$\begin{aligned} (V_{11} - V_{12}) (p_{2}-p_{1}) . \end{aligned}$$

Since $(V_{11} - V_{12}) > ( \alpha - \beta )$ it follows that the difference in fitness values of the two mechanisms reduces if behaviour becomes non-tightly linked with tracking states.

## Footnotes

1 For a recent discussion of the base-rate fallacy in debates concerning scientific realism, see Howson (2000), Magnus and Callender (2003) and Henderson (2015).

2 Similarly, the remaining fitness payoffs of the model need then to be introduced as $V_{11}^{*}=V_{11}P(S_{1})$ and $V_{22}^{*}=V_{22}P(S_{2})$.

3 Evolutionary game theory has recently been proposed as an alternative modeling framework for studying naturalistic theories of representation (Artiga 2016). For a link between evolutionary game theory and the models of evolution in stochastic environments employed in this paper, see McNamara (1995, 193–198).

4 I presume that Artiga is not referring to the false positive rate $P(T_{1}|S_{2})$ at this point. Assuming that the false positive rate exceeds 0.5 would be extremely high. It would amount to the cockroach tossing a coin that is biased towards triggering an evasive action in every situation in which predators are absent.

5 The distinction between the cases of idiosyncratic risk (or demographic stochasticity) and aggregate risk (or environmental stochasticity) is standard practice in biological discussions of evolution in variable environments (e.g., Gillespie 1973, 1974, 1977).

6 An alternative reading of response breadth contemplated by Sterelny (2003, 35, fn. 3) suggests that each organism has exactly one behavioural response given a tracking state. According to this reading, an increase in response breadth is an increase in the fineness with which the organism partitions its environment. Sterelny does not see a substantive difference between these two readings but opts for the idea that a behavioural response results from the combination of tracking states and other belief-like states capturing what else the organism notices and knows. In this paper I follow Sterelny’s choice on that subject matter.
